# Estimation of Crystalline Lens Material Properties From Patient Accommodation Data and Finite Element Models

**DOI:** 10.1167/iovs.64.11.31

**Published:** 2023-08-28

**Authors:** Andres de la Hoz, Eduardo Martinez-Enriquez, Susana Marcos

**Affiliations:** 1 Instituto de Óptica “Daza de Valdés,” Consejo Superior de Investigaciones Científicas (IO, CSIC), Madrid, Spain; 2 Center for Visual Science, The Institute of Optics, Flaum Eye Institute, University of Rochester, New York, United States

**Keywords:** crystalline lens, OCT, accommodation, FEM

## Abstract

**Purpose:**

The mechanical properties of the crystalline lens are related to its optical function of accommodation, and their changes with age are one of the potential causes for presbyopia. We estimated the mechanical parameters of the crystalline lens using quantitative optical coherence tomography (OCT) imaging and wavefront sensing data from accommodating participants and computer modeling.

**Methods:**

Full-lens shape data (from quantitative swept-source OCT and eigenlens representation) and optical power data (from Hartmann–Shack wavefront sensor) were obtained from 11 participants (22–30 years old) for relaxed accommodation at infinity and –4.5 D accommodative demand. Finite element models of lens, capsular bag, zonulae, and ciliary body were constructed using measured lens geometry and literature data, assuming a 60-mN radial force. An inverse modeling scheme was used to determine the shear moduli of the nucleus and cortex of the lens, such that the simulated deformed (maximally stretched) lens matched the participant's lens at –4.5 D.

**Results:**

The shear moduli of the nucleus and cortex were 1.62 ± 1.32 and 8.18 ± 5.63 kPa, on average, respectively. The shear modulus of the nucleus was lower than that of the cortex for all participants evaluated. The average of the two moduli per participant was statistically significantly correlated with age (*R*^2^ = 0.76, *P* = 0.0049).

**Conclusions:**

In vivo imaging and mechanical modeling of the crystalline lens allow estimations of the crystalline lens’ mechanical properties. Differences between nuclear and cortical moduli and their dependency with age appear to be critical in accommodative function and likely in its impairment in presbyopia.

The crystalline lens, a soft pliable lens located behind the iris, is responsible for one-third of the eye's focusing power, as well as for accommodation, the capacity to focus on objects at different distances. The crystalline lens is connected to the ciliary muscle via zonules attached to the lens’ peripheral and equatorial region; the expansion and contraction of the muscle can add or remove tension to the lens, altering its shape in the process. The crystalline lens consists of an external capsule, which encloses the softer, more deformable internal volume, which can be divided into two regions: a central nucleus and a peripheral cortex.

The ability to accommodate diminishes with age until it is fully lost, a condition called presbyopia, which affects the population after age 45 years. Various causes have been proposed as the drivers of presbyopia. The two most common theories are that presbyopia is caused by (1) age-related hardening of the internal crystalline lens material and (2) age-related changes in shape and size.^1^ Other factors, such as changes in the ciliary body and zonules, are believed to be less influential.

Therefore, the properties of the lens material are of interest in order to investigate the changes of the crystalline lens occurring in presbyopia, as well as the mechanisms of action of potential treatments.

Several crystalline lens properties, such as its low modulus, shape, and heterogeneity, make it unsuitable for traditional mechanical testing methods, such as uniaxial extensiometry. Therefore, other methods have been typically used to study mechanical properties of the crystalline lens. Fisher^2^ evaluated the stiffness of various crystalline lenses by spinning them and relating their deformation to the measured forces. Heys et al.^3^ and Weeber et al.,^4^ in separate works, used indentation methods on local regions of ex vivo crystalline lenses to evaluate the heterogeneous material properties of the lens. As with traditional mechanical testing methods, these are destructive techniques applied to ex vivo lenses and limited by the differences from physiologic conditions.

Noninvasive, nondestructive methods have also been applied to the study of the crystalline lens. Brillouin microscopy^5^^–^^7^ and ultrasound^8^^,^^9^ have been used on human and rabbit lenses to identify parameters that could be empirically or theoretically correlated to standard mechanical moduli (Young's or shear modulus).

In addition to the properties of the crystalline lens, the understanding of the process of accommodation and its decline with age is of fundamental interest. Mechanical models can shed light into the relations between mechanical parameters and geometrical and optical changes. These models are implemented using finite element approaches, which describe the lens by its geometry, discrete material properties, and boundary conditions (force or displacement loads). The structure deforms upon loading, and its strains, stresses, and displacements can be studied. The first major work in finite element modeling of the lens was by Burd et al.,^10^ who developed finite element models of three human lenses aged 11, 29, and 45 years, using experimental inputs from various sources. Over the years, various finite element models have been developed in order to evaluate the influence of various input parameters (such as zonular positions,^11^ shape,^12^ and age^13^) on the final output (stress distributions, power change, aberrations).

In recent years, finite element models and experimental techniques have been combined in what is generally called the inverse modeling approach. In this approach, two inputs are used for an objective function to be minimized. The first is the result of a measurement of a sample under loading and the second the result of a finite element model simulation of the same sample, in terms of both displacements and forces. The objective function is a comparison of these two inputs. The material properties in the finite element model are set as variables, and a minimization scheme is implemented to find the material properties that minimize the function. The final material coefficients are taken to represent the material's stiffness when described by a specific model.

The method has been used to estimate corneal mechanical parameters from dynamically deformed corneal images through application of an air pulse.^14^ Recently, it has been applied to the study of the crystalline lens: Reilly and Cleaver^15^ used an inverse method to estimate nuclear and cortical moduli from murine lenses subjected to compression from a piston. Wilde and colleagues^16^ combined Fisher's spinning lens method with an inverse model in order to estimate the mechanical properties of human lenses for various material models. In those works, the input used to evaluate the model consisted of ex vivo experimental measurements of deformations.

It is possible to apply inverse modeling techniques to the study of in vivo crystalline lenses. The process of accommodation involves subjecting the crystalline lens to a load, which can be characterized as a set of forces. Such forces have been quantified by various experimental and simulation studies.^17^^,^^18^ The change in accommodative states also allows for the quantification and definition of the solid in various states of loading (minimally and maximally deformed). With these data, an inverse model could retrieve valid constants for the material models used in the finite element model. One potential limitation is that most imaging techniques prevent the quantification of the entire lens, as the iris blocks the peripheral regions. Our group has recently proposed a method to reconstruct the full lens shape from the visible central region in anterior segment OCT images, the eigenlens method,^19^^,^^20^ which overcomes that limitation.

The purpose of this work was to build finite element models of crystalline lenses from patient data and simulate accommodation to estimate the mechanical properties of each lens.

## Methods

### Participants

Eleven young healthy volunteers participated in the study, with ages ranging from 22 to 30 years (mean age, 25.77 ± 2.5 years) and spherical refractive errors ranging from −6.75 to 0.75 D (mean refractive error, −2.04 ± 2.3). The protocols were approved by the institutional review board, and participants signed an informed consent form after they became familiarized with the nature and protocol of the study.

### Optical Coherence Tomography and Wavefront Sensing

A custom-developed swept-source system^21^ was used to image the participants. The system used a VCSEL swept-source (SL132120; Thorlabs, Newton, New Jersey, USA), centered at 1300 nm and a fiber-optic–based Mach–Zender interferometer configuration. The axial depth range was 15.95 mm in air, sampled by 1920 pixels, resulting in a pixel size of 8.3 µm, and the acquisition speed was 200,000 A-scans/s. Images were collected in one eye while the participant fixated at infinity and accommodated to a −4.5 D target. In addition, a custom-developed Hartmann–Shack aberrometer, coupled with the optical coherence tomography (OCT) system, consisting of a SuperLuminescent Diode (SLD-340-HP-850; Superlum, Carrigtwohill, Ireland) and wavefront sensor (WFS20-7AR/M; Thorlabs), was used to measure the wave aberrations in the relaxed and accommodating state (−4.5 D) in the same eye. In both systems, a Badal optometer was used to change the target vergence.

Each OCT image volume consisted of a stack of 150 images, in a raster scan of 300 A-scans/B-scan (covering a lateral range of 15 × 15 mm). The OCT images are automatically segmented and corrected for fan and optical distortion. Data then undergo the processes of segmentation, distortion correction, and tilt removal.^22^^–^^25^ The processed images have been used in a prior work.^20^

Wavefront measurements were performed automatically after the OCT acquisition and the Zernike polynomials provided by the wavefront sensor software were used to calculate the spherical equivalent using conversion formulas.^26^ The amplitude of accommodation was calculated subtracting the average spherical equivalent at each accommodative demand.

### Eigenlens Method

The eigenlens method was used to reconstruct the full lens shape from the data in the central region. The details of the method are described in previous publications.^19^^,^^20^ With this method, the full shape of the crystalline lens is represented by a series of crystalline lens forms, weighted by the eigenlens coefficients. The eigenlens is a very compact representation, as typically lenses can be represented with great accuracy (96%) with four to five eigenlens coefficients. From this estimation, each lens was described as a set of coordinates for 0 and 4.5 D states, to be used for a finite element model and subsequent minimization procedure.

### Finite Element Model

**Figure 1.**
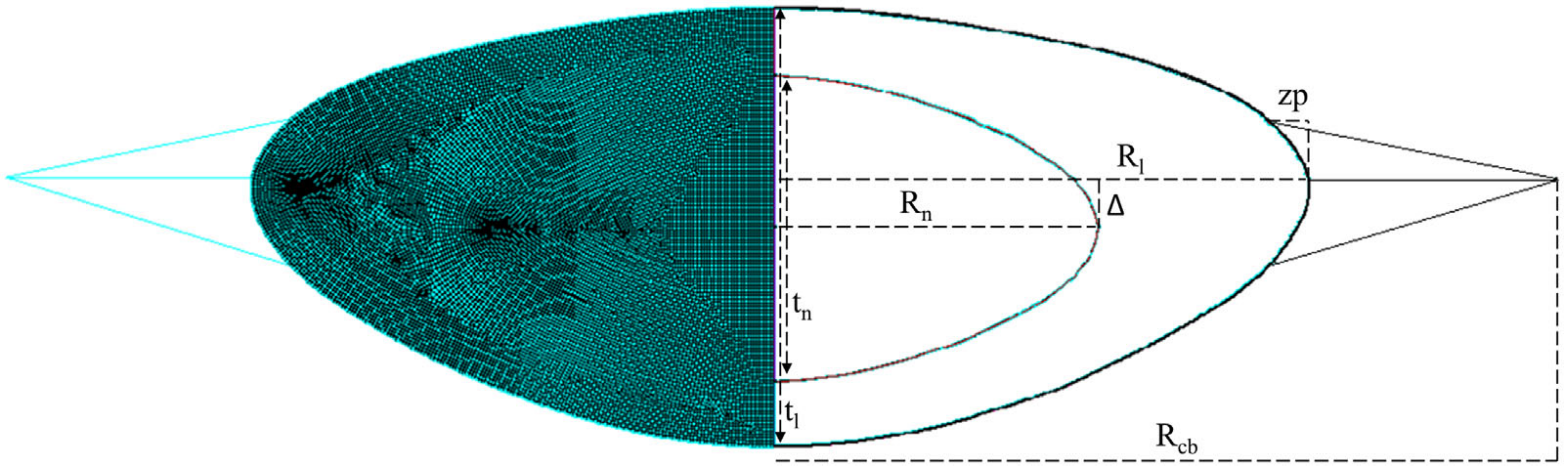
Standard lens model. *Right side* represents the finite element mesh, and *left side* represents lens dimensions. Lens radius (R_l_) and thickness (t_l_) are obtained from OCT experimental measurements and eigenlens reconstruction. Nucleus radius (R_n_) and thickness (t_n_) are set at 2.7 and 2.6 mm, with the center placed at an offset Δ of 0.5 mm. Zonule position (zp) and ciliary body radius (R_cb_) are determined from the literature as a function of age.

The accommodation model was created using ANSYS Mechanical APDL 20 (Canonsburg, Pennsylvania). The lens profile of the participant for a 4.5 D stimulus is used as the basis for the external geometry of the lens in its initial, undeformed state. The solid region of the model is divided into two parts: the cortex and the nucleus. The reconstructed profiles do not provide information about the size and shape of the nucleus. As there is a lack of consistent data on nucleus shape as a function of age or size^27^^,^^28^ in the literature, a constant nucleus size was used for all lenses in the model. The dimensions assigned were a thickness of 2.6 mm, a radius of 2.7 mm, and an offset Δ of 0.5 mm from lens equator to nucleus equator. The geometry is represented in Figure 1.

The nucleus and cortex of the lens are modeled using axisymmetric solid elements. The material model used is the neo-Hookean hyperelastic model. Under an assumption of incompressibility, this model has a single material parameter µ. As this parameter is closely related to the linear elastic shear modulus *G*, for the purposes of this article, the parameters for nucleus and cortex will be referred to as nuclear and cortical shear moduli, *G_n_* and *G_c_*. This hyperelastic model is selected to comply with the large deformation effects required for the problem. Although tissues and biological materials are often modeled with higher-order material models to account for highly nonlinear stress–strain behavior, simpler models have been the standard for crystalline lens simulation.^11^^–^^13^

The crystalline lens’ capsule was modeled using axisymmetric shell elements. The capsule is known to have variable properties and has sometimes been modeled with a variable thickness function. In order to limit the number of assumptions and extra variables in the model, a constant thickness of 13 µm was used.

The remaining model parameters were adapted from the work of Burd et al.,^10^ with the original sources being various experimental studies of accommodation and crystalline lens physiology. Young's modulus of the capsular bag was obtained from the relationship proposed by Krag et al.^29^ between lens capsule and age for lenses of participants under 35 years old:

(1) $$\begin{array}{c} E_{capsule}=0.03\;\times \left(age-35\right)+1.45\; \end{array}$$

The zonules were modeled as linear springs, with upper and lower zonules inserted into the external surface of the lens at a distance from the lens equator calculated from the following relationship, retrieved from the work of Farnsworth and Shyne^30^:

(2) $$\begin{array}{c} zp=\;R_{l}-\left(0.0311+0.0124\;\times age\right)\; \end{array}$$

The lens zonules were modeled using spring elements and assigned values of 1.2, 0.2, and 0.6 N/mm for anterior, central, and posterior zonules, respectively.

The final position of the zonules was determined by the following relationship (from Strenk et al.^31^):

(3) $$\begin{array}{c} R_{cb}=6.735-0.009\;\times age\; \end{array}$$

Loading in finite element accommodation models is typically represented by either a ciliary body displacement or a force. A ciliary body displacement would generate forces proportional to the material's elastic properties. As the goal of this work is to estimate those material properties, this load would not be appropriate. Therefore, a force load was used. The maximum force expected in the accommodation apparatus is approximately 80 mN.^17^ As participants in the experimental study did not have a full accommodative demand (4.5 D stimulus versus an expected average maximum of 6 D for the age range^32^), a force value below maximum, of 60 mN, was used in the finite model. This force is applied as a load on the node that connects all three zonules, with a vertical displacement restriction on the node.

The output of the finite element model includes the final coordinates (initial coordinates + displacement) of the nodes along the external surfaces of the crystalline lens.

### Inverse Model

A program was written in MATLAB (MathWorks, Natick, MA, USA) and used as the basis of an inverse model. First, a function calls ANSYS and solves the finite element model. The function then reads the solver output (the external surfaces of the lens represented by nodes and their coordinates) and compares it to the experimentally obtained surface of the lens at maximum deformation. The surfaces were evaluated for a pupil diameter of 6 mm. The comparison was done through a function *f_lens_*, defined as the root mean square of the difference between coordinates on the y-axis of experimental (*ye*) and simulated surfaces (*ys*) corresponding to values of x spaced at intervals of 100 µm.

(4) $$\begin{array}{c} f_{lens}\left(G_{n},G_{c}\right)=\;\sqrt{\frac{1}{n}\sum_{i=1}^{n}{\left(ys_{i}-ye_{i}\right)}^{2}}\; \end{array}$$

**Figure 2.**
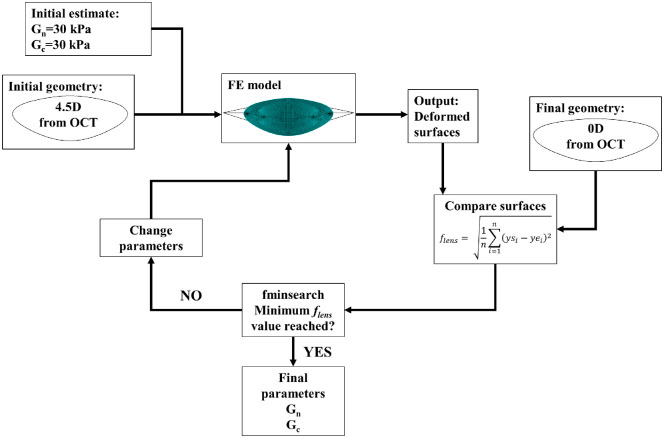
Inverse modeling procedure.

The *fminsearch* MATLAB function was used to minimize the *f_lens_* function. This is a multidimensional unconstrained nonlinear minimization using the Nelder–Mead algorithm. The initial parameter values were *G_n_*, *G_c_* = 30 kPa. For the purposes of this work, the only restriction in the minimization was that *G_n_*, *G_c_* > 0. The inverse model procedure is represented schematically in Figure 2.

### Lens Power

Simulated lens power was quantified using a virtual ray-tracing model built in ZEMAX (nine rays over 6-mm-radius pupil). The model uses a fixed refractive index of 1.42 and takes the anterior and posterior surfaces and thickness as its input to estimate the focal length of the system. The surfaces were first quantified using the MATLAB curve-fitting function to the equation that defines a standard surface in ZEMAX:

(5) $$\begin{array}{c} y=\;\frac{cx^{2}}{1+\sqrt{1-\left(1+k\right)c^{2}x^{2}}}\; \end{array}$$

where *c* is the curvature, *k* is the conic constant, and *x* is the coordinate in the radial axis.

### Model Evaluation

Additional simulations were evaluated in order to estimate effects of various parameters on the model. A grid evaluation for varying values of *G_n_* and *G_c_* was done for each lens in order to estimate the sensitivity of the function *f_lens_* to variations in these parameters. The sensitivity of the model to the constant or fixed force and stiffness parameters was assessed by minimizing the function *f_lens_* for a single lens and varying the modulus of the capsule (from 1 to 5 MPa), the spring constant of the zonules (from 0.1 to 0.8 N/mm), and the force applied in the accommodation process (from 10 to 80 mN).

The model of the lens as described by two regions with distinct material parameters (*G_n_*, *G_c_*) was compared to a model described by a single parameter *G*, by imposing the condition *G_n_* = *G_c_* on the minimization process.

## Results

The Table presents a summary of optimization results. The mean value for the nuclear modulus was 1.62 ± 1.32 kPa, and the mean value for the cortical modulus was 8.18 ± 5.63 kPa. For all lenses, the value of the nuclear modulus was lower than the value of the cortical modulus. The ratios of *G_c_*/*G_n_* varied, with seven of the lenses averaging a ratio of 3.83 ± 2.11. Two of the lenses (3 and 4) averaged much higher ratios (60.78 and 205.72). For two of the lenses, the value of *f_lens_* did not substantially decrease from its initial value. These lenses were not included in the results (see Discussion).

**Table.** Nuclear (*G_n_*) and Cortical (*G_c_*) Moduli Values Obtained From the Minimization Procedure and Corresponding *f_lens_* Value

Lens	Age, y	*f_lens_*, µm	*G_n_*, kPa	*G_c_*, kPa
1	22	11.61	0.78	5.11
2	22	8.26	1.78	4.04
3	24	14.93	0.14	8.51
4	26	16.42	0.11	22.63
5	26	26.93	2.68	4.59
6	26	22.37	1.07	6.76
7	27	14.40	1.70	8.15
8	29	29.88	1.97	7.02
9	30	10.90	4.31	6.78
Mean	25.77	17.25	1.62	8.18
SD	2.77	7.47	1.32	5.63

**Figure 3.**
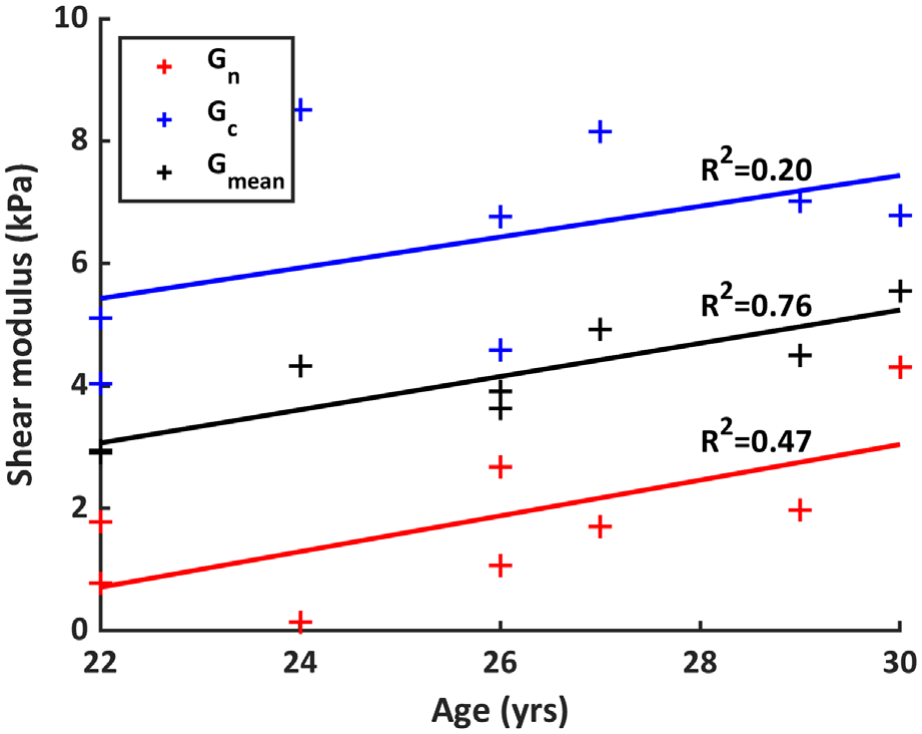
Nuclear, cortical, and average moduli as a function of age.

Figure 3 presents the relationship between age and moduli. For the purposes of this evaluation, lens 4 was removed from the calculation, on the basis of (a) largest deviations from the averages and much higher *G_n_*/*G_c_* ratio and (b) reduced sensitivity to *G_n_* (see Fig. 5). An average shear modulus value *G_mean_*, the mean of the lens’ nuclear and cortical moduli, was evaluated for all lenses. *G_mean_* correlates highly with age (*R*^2^ = 0.76, *P* = 0.0049). The relationship is weaker and not statistically significant for the nuclear (*R*^2^ = 0.47, *P* = 0.065) and cortical (*R*^2^ = 0.20, *P* = 0.25) moduli individually. The slope is similar for both nuclear and cortical moduli, indicating changes of 0.27 ± 0.02 kPa/y.

**Figure 4.**
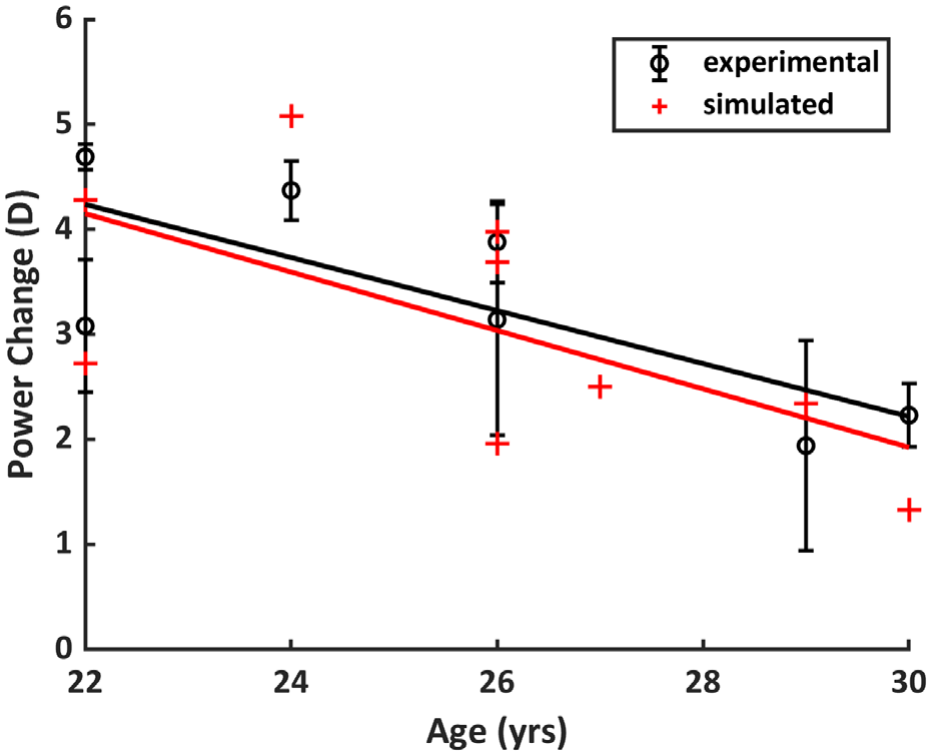
Experimental and simulated power change as a function of age.

Figure 4 compares the experimental and simulated (from ray tracing) refractive power age dependency. The relationship between age and refractive power is lower and, in the case of the simulated power, not statistically significant (*R*^2^ = 0.58, *P* = 0.034 for experimental and *R*^2^ = 0.40, *P* = 0.07 for simulated). The slopes on the plot are also similar, −0.25 (experimental) and −0.28 (simulated). The average simulated change in power (3.10 ± 1.22 D) is close to the average experimental power (3.33 ± 1.04 D). The average of the absolute difference between the lens-measured and lens-estimated power was 0.75 ± 0.55 D. For the experimental results, data for lenses 4 and 9 were unavailable.

## Discussion

### Sensitivity and Precision of the Model

**Figure 5.**
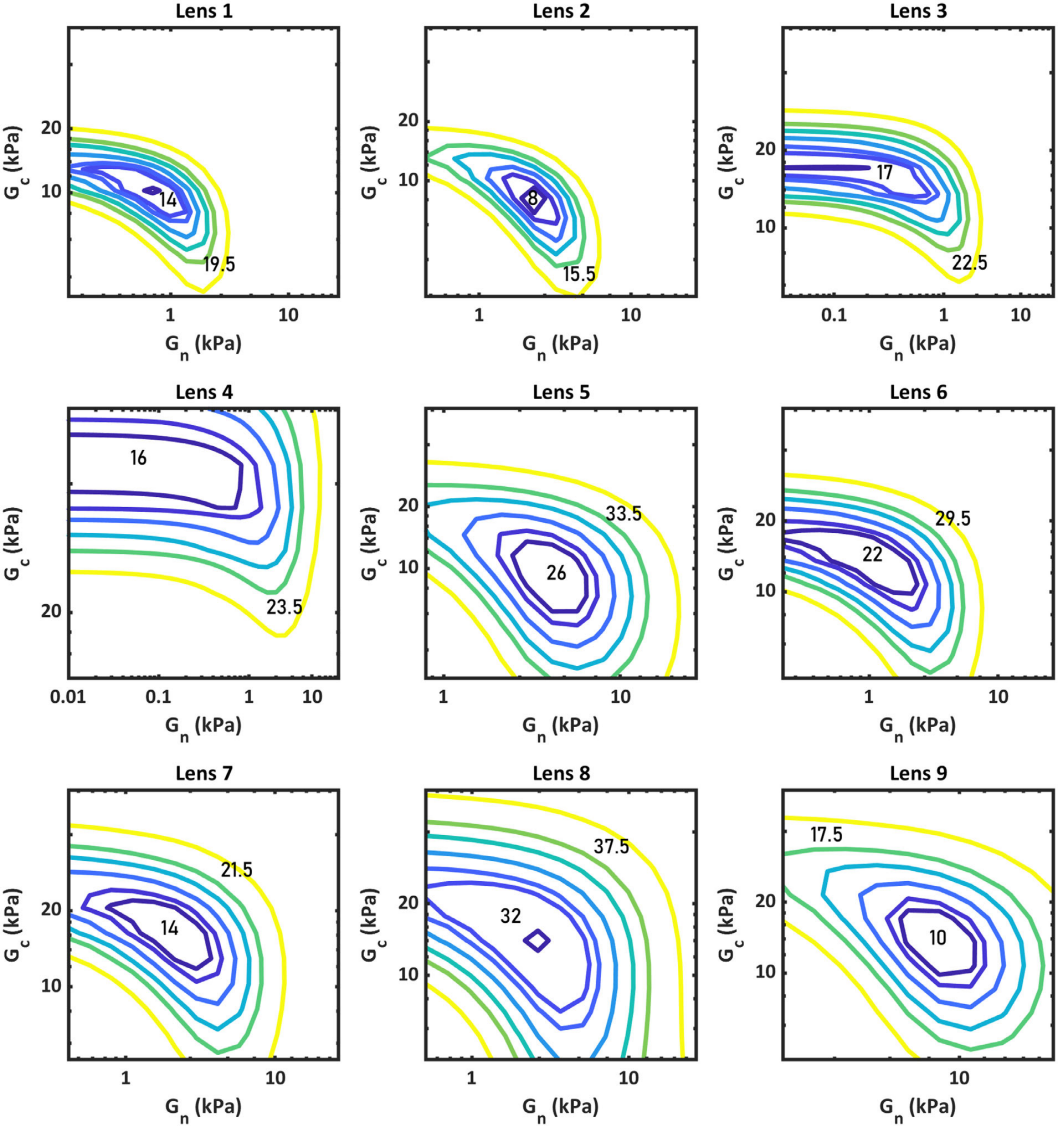
Relationship between the moduli and the value of function *f_lens_*.

The influence of the parameters of *G_n_* and *G_c_* on the function *f_lens_* was tested for each lens by evaluating a grid of values of the parameter generated around the values corresponding to the minimum *f_lens_* found in the minimization procedure. Figure 5 shows contour plots of the value of *f_lens_* in terms of *G_n_* and *G_c_* for lenses 1 to 9.

For each lens, there is a single minimum, indicating that the minimization function will not converge in one local minimum. The shape and size of the contours vary depending on the lens, indicating that the sensitivity of the function to the parameters is lens dependent. Lenses 3 and 4 show long contour bands on the *G_n_* axis for the values closest to the minimum. In these two cases, as the value of *G_n_* approaches zero, the finite element model becomes less sensitive to its value, and so does function *f_lens_*. This partly explains why these lenses yielded lower final *G_n_* values in the minimization process. This could be solved in various ways, the simplest one being to add boundaries to the minimization process in the form of limits to *G_n_* and *G_c_*, based on the existing literature and on sensitivity/precision values obtained from these models.

Optimizations were also evaluated for the specific condition where *G_n_* = *G_c_*. For this condition, the average value of *f_lens_* was 25.77 ± 9.57 µm compared to 17.25 ± 7.47 µm for the unrestricted model presented in the Table. The modulus with this restriction was also lower, 2.44 ± 1.17 kPa compared to the average modulus of 4.24 ± 2.25 kPa for the two-parameter model. The increase in *f_lens_* value indicates that the quality of the fit is poorer, and at least two distinct regions with different material properties are required to accurately model the lens’ deformation during accommodation.

### Influence of Nonoptimized Parameters

A number of parameters in the lens are held constant in each minimization procedure and can have an influence on the final outcome. The influence of these parameters was evaluated by testing the minimization procedure on lens 1 for a range of values of these parameters and recording the minimum *f_lens_* value and the corresponding average modulus *G_mean_*.

**Figure 6.**
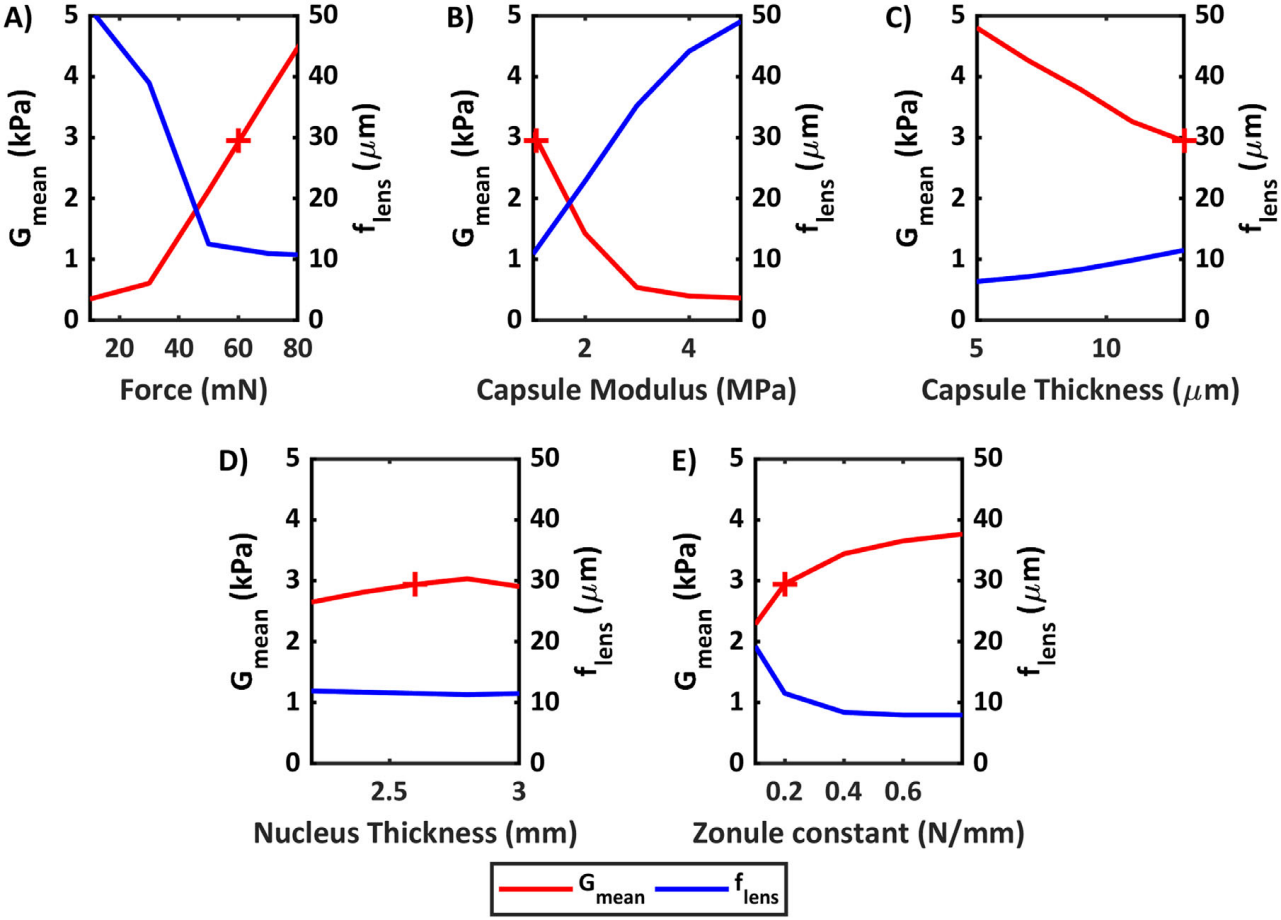
Effect of force boundary condition (**A**), capsular modulus (**B**), capsule thickness (**C**), nucleus thickness (**D**), and zonule constant (**E**) on average moduli and minimum objective function value obtained from optimization process. When value is constant, F = 60 mN, E_capsule_ = 1.06 MPa, and zonule constant = 0.2 N/mm. Average modulus results for these values are represented by a *red cross*.

Figure 6A shows the influence of the force boundary condition. For a range of force values from 10 to 80 mN, function *f_lens_* exponentially decreases in value from 42 µm for a force of 10 mN to a plateau of 12 µm for a force of 80 mN. The value of *G_mean_* obtained from the minimization procedure also increases with increasing force load. The interpretation of this result is that lower forces are not sufficient to deform the lens when accounting for capsular and zonular stiffness, even as *G_n_* and *G_c_* move toward zero. A similar effect is observed for the capsular modulus, represented in Figure 6B. The average shear modulus of the lens decreases with increasing capsular modulus, along with the value of *f_lens_*. Figure 6C shows the relationship between the capsule thickness and the estimated modulus. Per the slope of the graph, each additional micrometer of thickness decreases the average modulus by 0.225 kPa. The value of *f_lens_* was also found to decrease with decreasing capsular thickness. This could indicate that adjustments to the capsular thickness could improve the quality of the fit and minimization process. Although, in this case, the reduction in thickness improved the fit primarily in a region where the capsule is expected to be thickest.

Figure 6D shows the effect of altering the central thickness of the nucleus. The overall effects on the average modulus were low, but larger effects were seen in the nuclear modulus. The change from 2.2 to 2.6 mm of thickness resulted in an increase of 0.45 kPa in the estimated modulus of the nucleus. Figure 6E shows the effect of altering the zonule spring constants. Data are presented for equatorial zonule constant (with anterior and posterior zonule constants both being multiples of this number). The nuclear/cortical moduli increased with increasing spring constant and *f_lens_* decreased. These effects indicate that the fixed parameters of the model affect the values of the estimated material properties and also affect the quality of fit, limiting their range of likely values.

In the model, forces of 60 mN were assumed. Figure 6A shows that, beyond 50 mN, the value of *f_lens_* plateaus, indicating practically no further improvement in the quality of the fit. For forces below 50 mN, on the other hand, *f_lens_* is large and fit quality decreases. This suggests 50 mN is the lower boundary of the force during accommodation for these measurements. The upper boundary cannot be estimated from the change in *f_lens_*, but physiologically, the value is estimated to be around 80 mN.^17^ Since force and accommodation are directly related, this suggests that stimuli of at least 4 D are required to properly estimate lens mechanical properties from patient data, at least in this age range.

In the model, several parameters were assumed to have an age dependence (equations (1)–(3)). As a relationship was found between the age of the participant and the moduli obtained from the minimization process, an additional test was done to assess the influence of the age-dependent parameters. A fixed value for each of these parameter (capsular modulus, ciliary body radius, zonule position) based on the average age of the participants (25.77 years) was used, and the minimization procedure was repeated for all lenses. This did not result in any substantial difference in correlation between age and average moduli (*R*^2^ = 0.8) and resulted in an overall difference in average modulus of 0.23 kPa.

### Limitations of the Model and Minimization Procedure

For 2 of the 11 lenses evaluated, the minimization procedure was unable to reduce the value of *f_lens_* (final average value of 89 µm). These two lenses were not included in the results. The lenses were inspected for differences with respect to the entire set. The input data for lens geometry were evaluated, and it was found that the center of the lens, defined as the height at which the lens is at its maximum diameter, shifted 290 µm from the 0 D to 4.5 D geometry. The rest of the lenses in the sample had a much smaller shift (8 µm on average). This shift does not seem to reflect an axial shift of the entire lens during accommodation. The boundary conditions of the model, which restrict the axial shift of the zonules, seemingly prevented the lenses from adjusting to the expected shape.

To test this, the displacement of the axial shift in the zonules was changed from zero to an arbitrary value (100–150 µm), and then the minimization procedure was executed. After this, the value of *f_lens_* was minimized to 25 and 36 µm for each lens. These values are, nonetheless, in the higher range compared to the set of lenses presented in the results, with 36 µm being higher than any of the lenses presented in the Results section of this article. Average moduli values (*G_n_* = 0.22 kPa and *G_c_* = 9.6 kPa) were on the lower and higher ends of the averages. Results on these two lenses suggest that the modeling assumptions will not hold for every participant and that additional input may be required in some cases.

### Implications on the Understanding of the Mechanism of Accommodation

Comparing our results to those of previous publications can yield insights into the mechanisms of accommodation and presbyopia. The studies by Heys et al.,^3^ Weeber et al.,^4^ and Wilde et al.,^16^ using dynamic mechanical analyzers and a lens-spinning device, found smaller moduli values in the nucleus than in the cortex for lenses of pre-presbyopic participants, in agreement with our findings, although their moduli data were higher. Schachar et al.,^33^ on the other hand, found equal nuclear and cortical moduli for lenses of participants under 40 years of age. This is in contrast to our conclusions. Our results show that assuming equal values of nucleus and cortex significantly reduces the quality of the fit.

A lower modulus in the nucleus than in the cortex implies that the nucleus will deform more than the cortex. This is quantitatively supported by our simulations, which reveal an average change in nuclear and cortical thickness of 160 ± 42 µm and 49 ± 16 µm, respectively. Similar observations were qualitatively observed experimentally in imaging studies of the accommodating crystalline lens.^27^^,^^34^

Changes in the shape of the nucleus have been proposed before as crucial to accommodation.^35^ Parametric finite element modeling of the lens has shown the influence of a stiffness gradient to accommodation amplitudes.^36^ Our estimates of the material properties of the crystalline lenses in accommodating participants support the notion that a change in the nucleus’ shape and thickness is required for accommodation.

The lenses modeled in this work were in the range of 22 to 30 years and thus did not include presbyopes. We found a small increase of both moduli with age, similar to observations in presbyopic lenses in other studies (Weeber et al.,^4^ Wilde et al.,^16^ and Heys et al.^3^ for participants over 50, 45, and 30 years old, respectively). Larger differences are expected for a broader range of ages.

Heys et al.^3^ reported average shear moduli of 0.039 ± 0.014 kPa for the nucleus and 0.098 ± 0.0064 kPa for the cortex in participants younger than 30. Weeber et al.^4^ reported shear moduli of 0.023 kPa for the center of the lens and 0.2 kPa near the periphery for a 20-year-old. Wilde et al.^16^ reported a regression for shear moduli values, which yielded nuclear moduli of 0.06 kPa (22 years) to 0.10 kPa (30 years) and cortical moduli of 0.43 kPa (22 years) to 0.66 (30 years). Our estimated values are higher, at 1.62 (nucleus) and 8.18 (cortex) kPa for an average age of 25.6 years. Important differences in the estimated values can be expected due to the different methods (probe indentation, lens spinning, accommodation data) and experimental conditions (ex vivo versus in vivo, lens storage). For example, Wilde et al.^16^ reported approximately 2-fold increases in the shear modulus of the cortex (0.39 kPa to 0.93 kPa) from ages 20 to 60, whereas Weeber et al.^4^ found up to a 70-fold increase (0.2 to 14 kPa) from ages 20 to 60.

Our estimates are closest in magnitude to those reported by Wilde et al.,^16^ whose methodology most resembles ours (a finite element inverse method). The primary differences are that in our method, the capsular bag is present, which causes a stiffening effect. The assigned properties of the capsular bag (Figs. 6B, 6C) can affect the estimate. On the other hand, our method is estimated from in vivo data, which means storage conditions, strain rate, and other experimental factors are of less relevance.

A number of noninvasive approaches to evaluating the crystalline lens have been proposed in recent years, such as Brillouin microscopy^5^^–^^7^ and optical coherence elastography.^37^ These approaches have generally concluded that the nucleus is stiffer than the cortex for all ages, which contradicts our findings as well as those of Heys et al.,^3^ Weeber et al.,^4^ and Wilde et al.^16^ It should be noted, though, that some of these techniques have been used on nonaccommodating or presbyopic lenses, where a harder nucleus could be expected. Although this work refers to the shear modulus of the lens, it is important to note that this is not necessarily an intrinsic modulus. The choice to model the lens’ behavior in terms of a stress–strain constitutive relationship means that larger changes in shape will be represented by lower moduli. This is the standard assumption made in all finite element modeling of the crystalline lens. The lens’ macro- and microstructures could play an important role in its overall behavior, which might explain some of the conflicting findings from different techniques.

### Future Work and Improvements on the Model

The procedure described in this work to estimate material properties of the crystalline lens is inherently less direct than methods using ex vivo lenses, as the absolute values will be affected by the model's parameters. On the other hand, the procedure also presents several advantages, such as the avoidance of postmortem effects on the lenses, data relevant in physiologic conditions, and availability of participants. It can also be more easily extended to applications where individual anatomic and mechanical properties are needed, such as customized optomechanical modeling in the eye.

The lenses evaluated were from participants within a small age range (22–30 years). The estimated material constants were generally consistent and showed a small increase in the modulus with age. Future work will involve evaluating older participants in order to determine the correlation between age and moduli, as well as the maximum age at which the modeling approach can determine the moduli.

The force used in the accommodation model can affect the estimated values, as shown in Figure 6A. In this work, we have assumed a specific value (60 mN) and assumed the value is constant for all participants (reasonable given the age range). The differences in accommodative response for each participant and their relationship to age could suggest variations in the real force. Smaller forces for younger participants would decrease the estimate of the modulus by about 0.11 kPa/mN and slightly underestimate any existing age–modulus relationships.

Our results suggest that accommodative targets of at least 4 D are necessary for estimation of properties on participants in this age range. A higher accommodation target, or the maximum demand, is likely required to assess participants in a wider age range and to assess the relationship between age and the material properties. Intermediate steps would also provide valuable information here about nonlinear behavior.

Although the standard in finite element modeling of the crystalline lens is to treat the material's behavior as linear, the tissue's real constitutive relationships might be nonlinear. Intermediate steps could provide an avenue to assess whether material nonlinearity affects the response and, if so, whether the implementation of a different constitutive model would improve the model. We have made a preliminary attempt to estimate material properties using a nonlinear Ogden hyperelastic model and compare them to the neo-Hookean model by evaluating intermediate results (at 30 mN). The differences in the shape of the surfaces were low (*f_lens_* = 3.84 µm), indicating that nonlinear material effects might not be easy to detect in the deformed lens shape.

The nucleus was also shown to affect the estimated values, as shown in Figure 6D. It is possible to observe the outline of the nucleus with OCT imaging, although this was not the case for the data set used in this work. Implementing nucleus shapes determined from OCT images, ideally from the specific participant, could improve the accuracy of the estimation of the nuclear modulus.

Previous research on lens modeling has shown that varying the zonules (by changing the angles of insertion and direction of force loads) can affect the deformation of the lens and the corresponding surface curvature.^11^ Thus, it might be possible to improve the quality of the simulation–experimental fit by adjusting these parameters, although limitations may arise from including excessive degrees of freedom.

The eigenlens method, used to estimate the full volume of lenses in this work, can be used to describe full-lens geometry with a small number of coefficients. The method has also been extended to study the relationship between eigenlens coefficients and the age of the participant. This, along with the finite element approach used in this work, could be combined to develop a parametric model of lens accommodation. With such a model, it might be possible to quantify changes in shape and optical power as a function of input parameters such as material properties, shape coefficients, and age.

## Conclusions

A set of crystalline lens geometries from participants aged 22 to 30 years was evaluated in a finite element model of accommodation to estimate material properties. The estimated material properties were 1.62 ± 1.32 kPa shear modulus for the lens nucleus and 8.18 ± 5.63 kPa shear modulus for the lens cortex. Shear modulus of the cortex was higher than the nucleus for all lenses. The results suggest a relationship between the overall stiffness, the change in optical power, and the age of the participant. The magnitudes and trends of the data are in line with previous ex vivo mechanical studies of lens material properties and their relationship to age. The method presented uses in vivo patient data and, as such, shows promise for the development of broader accommodation simulation models that can account for influence of age, material, and other factors.
